# The complete mitochondrial genome of the feather mite *Trouessartia rubecula* Jablonska, 1968 (Astigmata: Analgoidea: Trouessartiidae)

**DOI:** 10.1080/23802359.2018.1476072

**Published:** 2018-05-25

**Authors:** Rocío Esteban, Jorge Doña, Joaquín Vierna, Antón Vizcaíno, David Serrano, Roger Jovani

**Affiliations:** a AllGenetics & Biology SL, A Coruña, Spain;; b Department of Evolutionary Ecology, Estación Biológica de Doñana (EBD-CSIC), Sevilla, Spain;; c Illinois Natural History Survey, Prairie Research Institute, University of Illinois at Urbana-Champaign, Champaign, IL, USA;; d Department of Conservation Biology, Estación Biológica de Doñana (EBD-CSIC), Sevilla, Spain

**Keywords:** Birds, ectosymbionts, host-symbiont, mitochondria

## Abstract

We assembled and annotated the complete mitochondrial genome of *Trouessartia rubecula*, the first feather mite complete mitochondrial genome from the largest feather mite superfamily Analgoidea (ca. 1150 spp). The mitogenome was composed of 13 protein, 17 tRNA, and 2 rRNA-coding genes and was 14,125 bp in length.

Feather mites (Acariformes: Astigmata: Analgoidea and Pterolichoidea) are the most common, abundant, and diverse ectosymbionts of birds (Doña et al. 2016). *Trouessartia rubecula* is a feather mite species which inhabits the flight feathers of European robins *Erithacus rubecula* (Doña et al. 2016). In this study, we present the complete mitochondrial genome of *T. rubecula*, which is the first feather mite complete mitochondrial genome from the superfamily Analgoidea.

Total genomic DNA was extracted using the MicroSpin kit (Real) from 30 *T. rubecula* individuals sampled from a single individual of *E. rubecula* at Corterrangel (Huelva, Spain) (37°56′14.1″N, 6°36′00.2″W). The DNA sample was submitted to the Novogene Bioinformatics Institute (Beijing, China) for library preparation and sequencing in a lane of an Illumina HiSeq 4000 PE150.

After performing a quality filtering step with Trimmomatic 0.33 (Bolger et al. 2014), the reads were de-novo assembled using ABySS 2.0.2 (Simpson et al. 2009). A 14.38 kb contig which showed 77% of nucleotide identity to the mitochondrial genome of *Dermatophagoides pteronyssinus* (GenBank accession number: EU884425.1) was found.

The MITObim software 1.9 (Hahn et al. 2013) was used to verify the reconstructed sequence using 2500 bp of the ABySS contig as seed. A contig of 14.59 kb was obtained and circularized using the script circules (https://github.com/chrishah/MITObim). Finally, the COI gene was placed at position 0 using Geneious 10.2.2 (Kearse et al. 2012).

The MITObim contig was kept for downstream analyses (GenBank accession number: MH208456). The final length of the mitochondrial genome was 14.13 kb.

MITOS 2 (Bernt et al. 2013) was used to annotate protein, tRNA, and rRNA-coding genes. The protein-coding regions were manually validated using the ORFfinder tool (https://www.ncbi.nlm.nih.gov/orffinder/).

The mitogenome was composed of 13 protein, 17 tRNA, and 2 rRNA-coding genes. The 12S rRNA and 16S rRNA genes were 623 and 680 bp, respectively. The base composition was 28.53% A, 44.74% T, 10.24% C, and 16.49% G. The protein-coding sequence length was 10,797 bp, encoding 3599 amino acids.

Most of the tRNA coding genes showed TV-replacement loops and their lengths varied from 53 bp to 61 bp (Klimov and OConnor 2009). tRNA-Ala, tRNA-Glu, tRNA-Ile, tRNA-Tyr, and tRNA-Val genes could not be predicted. Although the lack of certain tRNA coding genes has been previously observed in the Acaridae family (Yang and Li 2015), further research will be needed for a better reconstruction of the tRNAs of *T. rubecula*.

A maximum-likelihood phylogeny was inferred (Figure 1). In brief, we downloaded all available whole mitochondrial genomes of astigmatan mite species (plus an outgroup from Mixonomata) from the NCBI GenBank database (accession date: 2 April 2018). Mitochondrial genomes were aligned using MAFFT v7.222 (Katoh et al. 2002), and the alignment was trimmed using Trimal v1.4 (Capella-Gutiérrez et al. 2009). We inferred the tree using IQ-TREE (Nguyen et al. 2015) and ModelFinder (Kalyaanamoorthy et al. 2017) was used to find the optimal evolution model. Overall, the phylogenetic relationships found in this study were congruent with previous studies on the phylogeny of these mites (Klimov and OConnor 2013).

**Figure 1.**
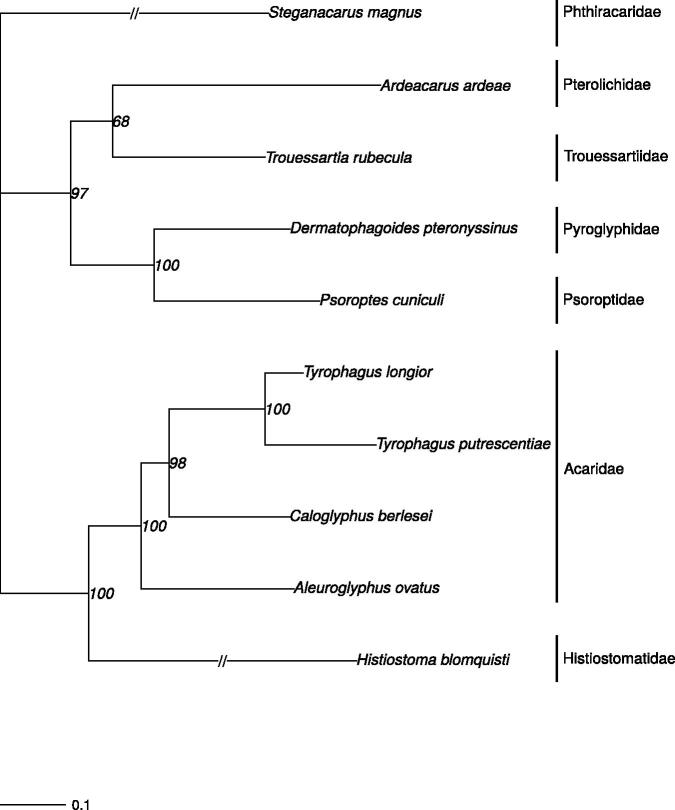
Phylogram based on the mitogenome sequences of *Trouessartia rubecula* (MH208456; this study) and eight other Astigmata mites (plus an outgroup from Mixonomata). The following mitochondrial genomes were used (accession numbers are in parentheses): *Tyrophagus longior* (NC_028725), *Tyrophagus putrescentiae* (NC_026079), *Ardeacarus ardeae* (KY352304), *Dermatophagoides pteronyssinus* (EU884425), *Psoroptes cuniculi* (NC_024675), *Caloglyphus berlesei* (NC_024637), *Aleuroglyphus ovatus* (KJ571488), *Histiostoma blomquisti* (NC_031377), and *Steganacarus magnus* (NC_011574), which was used as outgroup (Dabert et al. 2010). The phylogenetic tree was estimated from 500 bootstrap (BS) replicates in IQ tree. BS support values are indicated at each node and the scale bar indicates nucleotide substitutions per site.
